# Clinical impact of timing of surgery on outcomes in preterm infants with surgical necrotizing enterocolitis

**DOI:** 10.21203/rs.3.rs-3084887/v1

**Published:** 2023-06-26

**Authors:** Parvesh Mohan Garg, Robin Riddick, Md Abu Yusuf Ansari, Isabella Pittman, William Hillegass

**Affiliations:** Wake Forest University; University of Mississippi Medical Center; University of Mississippi Medical Center; University of Mississippi Medical Center; University of Mississippi Medical Center

**Keywords:** Surgical Necrotizing Enterocolitis, Neonate, Timing of disease onset, Outcomes, Preterm infants, Laparotomy, Penrose Drain

## Abstract

**Background:**

The clinical impact of the timing of surgery on outcomes in preterm infants with surgical necrotizing enterocolitis (NEC) is not well defined.

**Aim:**

We sought to investigate the impact of the different timing of surgery from the day of NEC diagnosis on clinical outcomes in preterm infants with surgical NEC.

**Study Design:**

Retrospective Cohort Study.

**Subjects:**

Preterm 75 infants admitted between January 2013 and December 31, 2018, with an NEC (Bell stage III) diagnosis.

**Outcomes:**

Comparison of clinical information by the timing of surgery at three different time points (less and more than 48 hours, 96 hours, and 168 hours) in preterm infants with surgical NEC.

**Results:**

75 infants were included in the analysis. Those who received surgery after 48 hours (n= 29/75) had lower median gestational age, lower birth weight, had less pneumoperitoneum, were out born less frequently, had higher acute kidney injury, were intubated and ventilated more frequently, and had higher hemorrhagic and reparative lesions on histopathology than those receiving surgery after 48 hours. Infants receiving surgery after 96 hours had similar trends expect had significantly lower hematocrit and more prolonged parenteral nutrition dependence than less than 96 hours group. The infants receiving surgery after one week had significantly lower birth weight and had higher reparative changes and cholestasis than those receiving surgery < 1 week.

There was no significant impact of surgery timing on the length of bowel loss, surgical morbidity, Bronchopulmonary dysplasia, white matter injury, and mortality.

**Conclusion:**

The infants receiving surgery later were young and smaller and received parenteral nutrition longer with no significant impact on morbidities and mortality. Our data point out that there are advantages of operating early with fewer morbidities which need further confirmation and evaluation in large multicentric prospective studies or clinical trials.

## Introduction

Necrotizing enterocolitis (NEC) affects 3–10% of very low birth weight infants (1, 2). NEC is a leading cause of morality and morbidities among preterm neonates, leading to increased health care costs. Those infants with NEC who progress to bowel perforation are managed with either peritoneal drainage (PD) or laparotomy (LAP). The published randomized control trials (3–5) comparing the Penrose drain and laparotomy did not report any duration of failed medical management, timing of surgery following NEC onset or the impact of intervention (PD/LAP) on the systemic morbidities such as bronchopulmonary dysplasia, acute kidney injury or white matter brain injury on brain MRI (5).

Most of the NEC infants receiving surgical care are initially managed with medical treatment. However, the duration of medical treatment varies among different surgical centers as directed by on-call surgeon. Patients not responding to the medical treatment have been associated with delayed surgery, leading to longer parenteral nutrition dependence and higher morality (6) indicating the impact of timing of surgical invention on clinical outcomes in preterm infants with NEC/SIP. Hence, the clinical impact of timing of surgery on outcomes in preterm infants with surgical necrotizing enterocolitis (NEC) are not well defined and the optimal time of surgical intervention is still an open question.

In this report, we sought to investigate the impact of different timing of surgery from the day of NEC diagnosis on clinical outcomes in preterm infants with surgical NEC. Our primary hypothesis is that preterm infants with different timing of surgery would have different clinical outcomes. In our NEC cohort study, we did retrospective comparison of clinical information by the timing of surgery at three different time points (less and more than 48 hours, 96 hours and 168 hours) in preterm infants with surgical NEC.

## Methods

The study was conducted at a level 4 NICU at the University of Mississippi Medical Center (UMMC) after the Institutional Review Board approval. Our unit admits around 1000 infants annually including referrals from the surrounding counties. The UMMC Institutional Review Board approved this retrospective study with a waiver of informed parental consent. All infants admitted between January 2013 and December 31, 2018 with an surgical NEC (Bell stage III)/SIP diagnosis were included in the study (7). In our study, we had 97 cases, including 75 cases of surgical NEC and 22 cases of SIP. Preterm infants diagnosed with congenital heart disease, medical NEC, and intestinal atresia were excluded. The cohort derivation is summarized in **Fig. 1.**

### Clinical Information

Maternal information collected included chorioamnionitis, antenatal steroids, and pregnancy-induced hypertension (PIH). We collected demographic variables including birth weight, gestational age, Apgar scores, delivery mode, small for gestational age, sex, race, and the out born status We recorded clinical data including respiratory support, inotrope need 24 hours following NEC, patent ductus arteriosus (PDA), ibuprofen /indomethacin treatment, blood culture information sent at the time of NEC onset, antibiotic used, and the frequency of cholestasis (direct bilirubin ≥ 2 mg/dl) following NEC.

### NEC information

NEC was defined using Bell’s criteria(7), Bell stage III/surgical NEC frequency was gathered(7). In addition, we recorded information on the age at NEC diagnosis and diagnosis of NEC was made on abdominal X-ray findings.

SIP infants had pneumoperitoneum on Xray, or intestinal perforation noted on intestinal pathology and less than five centimeters of bowel resected with no necrosis or inflammation on the histopathology. At our center, infants with birth weight less than 1 kg with pneumoperitoneum and critically sick were managed with PD first and later received laparotomy if needed.

### Histopathological information

Hematoxylin & eosin-stained intestinal tissue sections were evaluated by a pathologist for inflammation, necrosis, inflammation, hemorrhage, and reparative changes and were classified as - grade 1 was limited to the mucosa, grade 2 changes extended to the submucosa, grade 3 to the muscularis, and grade 4 change was transmural.

### Depth Evaluation

A score of 0 was assigned when the exam appeared normal, 1 for 1–25% necrosis/inflammation, 2 when 25–50% area involved, 3 when 50–75% area was affected, and 4 when > 75% changes were seen(8). The reparative changes included the presence of fibroblasts, epithelial regeneration and neovascularization.

SIP infants had pneumoperitoneum on Xray, or intestinal perforation noted on intestinal pathology and less than five centimeters of bowel resected with no necrosis or inflammation on the histopathology. At our center, infants with birth weight less than 1 kg with pneumoperitoneum and critically sick were managed with PD first and later received laparotomy if needed.

### Bronchopulmonary dysplasia (BPD) data

BPD was categorized as mild, moderate, and severe based on the oxygen requirement at assessment at 36 weeks of corrected gestational age (9). We also recorded information on the type of steroids used following the NEC diagnosis.

### Kidney Function Data

The Modified Neonatal Staging Criteria described in Improving Global Outcomes (KDIGO) Clinical Practice Guideline for AKI was used to assess the kidney injury (10–14). We examined daily urine output and all the serum creatinine measurements the day before NEC diagnosis and up to seven days following NEC diagnosis.

### Outcome Data

Post-operative information such as parenteral nutrition days, time to full feeds (≥ 120 ml/kg/day), post-operative ileus days, length of stay, surgical morbidities and the death were measured. We defined mortality as death due to any reason before hospital discharge. Neonates receiving the parenteral nutrition for > 90 days were classified in the intestinal failure category. Surgical morbidity was classified as wound dehiscence, surgical site infections (including abscesses), strictures, fistulas, adhesions, and perforations.

### Brain Injury Information

Brain MRI without contrasts obtained at the term equivalent age and scored independently by two pediatric radiologists using a Woodward et al. scoring system of eight scales for white and gray matter injury (15).

### Statistical Methods

Demographic and clinical factors in infants with surgical NEC and NEC/SIP cohorts were compared based on the timing of the surgical intervention. Continuous data were summarized as median (1st quartile, 3rd quartile) with Mann-Whitney U or Kruskal-Wallis tests for differences. Categorical data were expressed as counts and percentages, with group differences assessed by Chi-squared or Fisher’s exact tests. All analyses were performed in R statistical software (version 4.2.1), and a *p*-value < 0.05 was considered statistically significant.

## Results

### NEC Cohort:

The data are summarized in Tables 1–4.

Seventy-five infants were included in the analysis. Those who received surgery after 48 hours (n = 29/75) had lower median gestational age (26 weeks [24.4;27.3] vs. 27.8 [25.4;31.6]; p = 0.013), lower median birth weight (690 grams [620;920] vs. 1005 [780;1778]; p = < 0.001), had less pneumoperitoneum (24% vs. 50%,p = 0.047), were outborn (51.7% vs. 78.3%;p = 0.032) less frequently, had higher acute kidney injury (22 (75.9%) vs. 22 (47.8%); p = 0.031), were intubated and ventilated more frequently (p = 0.025) and had higher hemorrhagic (P = 0.019) and reparative lesions (p = 0.004) on histopathology than those receiving surgery less than 48 hours.

Infants receiving surgery after 96 hours had similar trends except had significantly lower hematocrit (33.3 [27.6;36.8] vs. 37.1 [30.6;40.]; p = 0.041) and longer parenteral nutrition dependence (109 days [76.0;147] vs. 65.0 [22.0;11]; p = 0.011) and more adhesions (19.2% vs.4.08%); p = 0.045) than the infants receiving surgical invention at less than 96 hours.

The infants receiving surgery after one week had significantly higher reparative changes (p = 0.013) and cholestasis (88.9% vs.59.2%; p = 0.045) than those receiving surgery < 1 week.

There was no significant difference in the length of bowel loss, bronchopulmonary dysplasia and white matter injury, length of stay, and mortality in preterm infants with surgical NEC receiving intervention at three different time points.

### NEC + SIP Cohort

97 infants were included in the analysis. We assessed the outcomes in the combined cohort at the 96 hours cutoffs. 63/96 (64.9%) infants received surgical invention before 96 hours, and 34 (35.4%) infants received surgical therapy after 96 hours following the NEC onset.

Infants receiving surgical invention > 96 hours had significantly lower gestational age (25.5 weeks [24.0;26.9] vs. 27.0 [25.0;31.3]; p = 0.006), had lower birth weight (687 grams [600;902] Vs. 940 [710;1495]; p < 0.001), had less often pneumoperitoneum on the abdominal x-ray (29.4% vs. 57.1%, p = 0.017), had hemorrhagic (p = 0.04)and reparative (p = 0.003) lesions more often on the intestinal histopathology, had patent ductus arteriosus more often (76.5% vs. 50.8%, p = 0.02), needed assisted ventilation more frequently ( p = 0.013) and received parenteral nutrition for a longer duration 112 days [76.5;145] vs. 65.0 [23.0;112],p = 0.004) following surgery compared to the infants receiving surgical invention before 96 hours.

There was no significant impact of timing surgery on the length of stay, surgical morbidities, or mortality in the two groups. The data are summarized in **Supplemental Table 1.**

## Discussion

Our study is one of the first reports evaluating the impact of the different timing of surgery from the day of NEC diagnosis on clinical outcomes in preterm infants with surgical NEC comprehensively. Our data suggests infants receiving surgery at later time points following the disease onset were younger and smaller compared to those receiving surgery earlier. Infants receiving surgery after 48 hours were sicker, as evidenced by the higher hemorrhagic lesions on the intestinal histopathology and had AKI. Infants are most likely operated on later because surgeons give a full chance for medical treatment to sick infants before saying yes to surgical intervention. Those receiving surgery later were most likely to receive parenteral nutrition for a longer duration and had cholestasis more frequently than infants receiving surgery earlier, most likely due to delay in starting enteral feeds and reaching full feeds. Additionally, those infants operated on after 96 hours following surgical NEC had more adhesions following disease onset and later received another surgery. However, we did not observe any significant impact of the timing of surgery on the length of hospitalization, other systemic morbidities, including BPD, white matter injury on brain MRI and mortality.

Only a few studies report this aspect of surgical clinical care and outcomes; however, a recent population-based, prospective, population-based, observational study of all 27 pediatric surgical hospitals reported that infants not responding to the medical management lead to 30 hours delay in surgical invention and was most likely associated with longer duration of parenteral nutrition or mortality at 4 weeks following the NEC surgery(6). In our cohort, we observed infants operated on after 96 hours or four days following NEC onset received parenteral nutrition for more than 45 days or six weeks. We did not observe any significant difference in overall mortality in this cohort; however, the infants receiving surgery in less than 48 hours trended toward 12% lower mortality (28 .3% vs. 41.45, P = 0.36). However, infants operated on later had higher perioperative morbidity than at earlier time points.

The randomized control trials to date do not observe a significant difference in the duration of parenteral nutrition or mortality. However, the timing of the surgical intervention relative to the NEC diagnosis was not reported (3, 4). Tepas et al. reported the relationship between the seven metabolic derangements (positive blood culture, acidosis, bandemia, thrombocytopenia, hyponatremia, hypotension, or neutropenia) following NEC onset and clinical decision-making of intervention and the observation (16). In their cohort, the appearance of any 3 of 7 metrics indicated timely operative intervention, and the mean time between the initial intervention (observation vs. intervention) and evaluation was two days.

Our study’s strengths include a detailed and comprehensive analysis of impact of the timing of surgery of the clinical outcomes in preterm infants with surgical NEC.

The limitations of our study included, First, this was a single-center experience, reducing the study’s generalizability. Most of the infants with NEC were African American in our cohort, partly due to race distribution in Mississippi. Second, sample size limits our power to detect associations between clinical risk factors and outcomes in preterm infants with surgical NEC and the multiple comparisons may result in type I errors.

In conclusion, the preterm infants receiving surgery later were smaller, younger, had higher hemorrhagic lesions, had acute kidney injury in the perioperative period, were most likely to receive parenteral nutrition for a longer duration, and had more frequent cholestasis than infants receiving surgery earlier. However, However, we did not observe any significant impact of the timing of surgery on the length of hospitalization, other systemic morbidities, including BPD, white matter injury on brain MRI and mortality. Our data point out that there are advantages of operating early with fewer morbidities which need further confirmation and evaluation in large multicentric prospective studies or clinical trials.

## Figures and Tables

**Table 1:** Demographics, Prenatal and Clinical Information in infants with NEC at < or > 48, 96, 168-hour time points

	48 hours					96 hours			168 Hrs		
	n	n = 75	≤ 48 hours n = 46	>48 hours n = 29	*p*	≤96 hours n = 49	>96 hours n = 26	*p*	≤168 hrs n = 57	>168 hrs n = 18	*p*
**Prenatal Information**											
Pregnancy Induced Hypertension, n (%)	75	21 (28.0)	9 (19.6)	12 (41.4)	0.07	12 (24.5)	9 (34.6)	0.51	13 (22.8)	8 (44.4)	0.14
Chronic Hypertension, n (%)	65	12 (18.5)	7 (17.5)	5 (20.0)	0.99	7 (16.7)	5 (21.7)	0.74	8 (16.3)	4 (25.0)	0.47
Chorioamnionitis, n (%)	74	4 (5.41)	2 (4.44)	2 (6.90)	0.64	2 (4.17)	2 (7.69)	0.61	4 (7.14)	0 (0.00)	0.57
Maternal Inflammatory Response, n (%)	21	14 (66.7)	6 (85.7)	8 (57.1)	0.34	7 (87.5)	7 (53.8)	0.17	11 (84.6)	3 (37.5)	0.06
Fetal Inflammatory Response, n (%)	21	9 (42.9)	3 (42.9)	6 (42.9)	0.99	4 (50.0)	5 (38.5)	0.67	7 (53.8)	2 (25.0)	0.37
Maternal Vascular under perfusion, n (%)	21	12 (57.1)	4 (57.1)	8 (57.1)	0.99	5 (62.5)	7 (53.8)	0.99	9 (69.2)	3 (37.5)	0.2
SGA, LGA Placentas, n (%)	14	3 (21.4)	0 (0.00)	3 (37.5)	0.21	0 (0.00)	3 (37.5)	0.21	0 (0.00)	3 (60.0)	**0.028**
Antenatal Steroids, n (%)	72	51 (70.8)	27 (62.8)	24 (82.8)	0.12	30 (65.2)	21 (80.8)	0.26	37 (68.5)	14 (77.8)	0.65
**Infant Demographics**											
Small for Gestational Age, n (%)	75	22 (29.3)	11 (23.9)	11 (37.9)	0.3	12 (24.5)	10 (38.5)	0.32	15 (26.3)	7 (38.9)	0.47
Male, n (%)	75	51 (68.0)	32 (69.6)	19 (65.5)	0.91	35 (71.4)	16 (61.5)	0.54	40 (70.2)	11 (61.1)	0.67
Ethnicity, n (%)	75				0.9			0.99			0.45
African American		55 (73.3)	33 (71.7)	22 (75.9)		36 (73.5)	19 (73.1)		43 (75.4)	12 (66.7)	
Caucasian		18 (24.0)	12 (26.1)	6 (20.7)		12 (24.5)	6 (23.1)		13 (22.8)	5 (27.8)	
Other		2 (2.67)	1 (2.17)	1 (3.45)		1 (2.04)	1 (3.85)		1 (1.75)	1 (5.56)	
Gestational Age (weeks, median [IQR])	75	26.6 [25.0;30.3]	27.8 [25.4;31.6]	26.0 [24.4;27.3]	**0.013**	27.6 [25.4;31.6]	25.8 [24.4;26.9]	**0.01**	27.0 [25.1;31.1]	26.4 [24.6;27.4]	0.29
C-section, n (%)	75	49 (65.3)	29 (63.0)	20 (69.0)	0.78	31 (63.3)	18 (69.2)	0.79	37 (64.9)	12 (66.7)	0.99
Birth Weight (g, median [IQR])	75	920 [670;1380]	1005 [780;1778]	690 [620;920]	**<0.001**	1000 [740;1621]	690 [635;902]	**0**	940 [670;1540]	730 [610;942]	0.06
Apgar score < 6 at 5 min, n (%)	73	20 (27.4)	10 (22.2)	10 (35.7)	0.32	10 (20.8)	10 (40.0)	0.14	16 (28.6)	4 (23.5)	0.77
Out born, n (%)	75	51 (68.0)	36 (78.3)	15 (51.7)	**0.032**	38 (77.6)	13 (50.0)	**0.03**	41 (71.9)	10 (55.6)	0.31
**Infant Medical Information Prior to NEC**											
Patent Ductus Arteriosus, n (%)	75	42 (56.0)	22 (47.8)	20 (69.0)	0.12	23 (46.9)	19 (73.1)	0.05	30 (52.6)	12 (66.7)	0.44
PDA Surgical Ligation, n (%)	75	2 (2.67)	1 (2.17)	1 (3.45)	0.99	1 (2.04)	1 (3.85)	0.99	2 (3.51)	0 (0.00)	0.99
Indomethacin Use, n (%)	75	9 (12.0)	4 (8.70)	5 (17.2)	0.3	4 (8.16)	5 (19.2)	0.26	7 (12.3)	2 (11.1)	0.99
Blood Transfusion Before NEC, n (%)	60	57 (95.0)	37 (94.9)	20 (95.2)	0.99	39 (95.1)	18 (94.7)	0.99	43 (93.5)	14 (100)	0.99
Hematocrit Before NEC (median [IQR])	54	34.7 [29.6;38.5]	37.1 [30.2;41.7]	33.9 [27.8;37.0]	0.08	37.1 [30.6;40.9]	33.3 [27.6;36.8]	**0.04**	35.2 [29.8;39.8]	34.5 [27.7;37.4]	0.42
Platelet Transfusion Prior to NEC, n (%)	73	60 (82.2)	36 (81.8)	24 (82.8)	0.99	37 (78.7)	23 (88.5)	0.36	44 (80.0)	16 (88.9)	0.49
Blood Transfusion before NEC, n (%)	53	11 (20.8)	7 (21.2)	4 (20.0)	0.99	7 (20.6)	4 (21.1)	0.99	7 (17.5)	4 (30.8)	0.43

Categorical variables are presented as count (column percentage). The presence of bold values signifies p < 0.05. Continuous variables are presented as mean (standard deviation) if normality has been met and, if not normally distributed, as median with interquartile range.

**Table 2:** NEC clinical and histopathology information

	48 hours					96 hours			168 hours		
	n	n = 75	# x2264; 48 hours n = 46	> 48 hours n = 29	*p*	≔ 96 hours n = 49	> 96 hours n = 26	*p*	≤ 168 hrs n = 57	> 168 hrs n = 18	*p*
**NEC Disease Features**											
Clinical Presentation of NEC, n (%)	75				0.81			0.99			0.99
Abdominal Distension		67 (89.3)	40 (87.0)	27 (93.1)		43 (87.8)	24 (92.3)		50 (87.7)	17 (94.4)	
Bloody Stools		7 (9.33)	5 (10.9)	2 (6.90)		5 (10.2)	2 (7.69)		6 (10.5)	1 (5.56)	
Feeding Intolerance		1 (1.33)	1 (2.17)	0 (0.00)		1 (2.04)	0 (0.00)		1 (1.75)	0 (0.00)	
Radiological Findings, n (%)											
Pneumatosis	75	39 (52.0)	26 (56.5)	13 (44.8)	0.45	28 (57.1)	11 (42.3)	0.33	34 (59.6)	5 (27.8)	**0.037**
Portal Venous Gas	75	7 (9.33)	6 (13.0)	1 (3.45)	0.24	6 (12.2)	1 (3.85)	0.41	7 (12.3)	0 (0.00)	0.19
Pneumoperitoneum	75	30 (40.0)	23 (50.0)	7 (24.1)	**0.047**	25 (51.0)	5 (19.2)	**0.015**	27 (47.4)	3 (16.7)	**0.041**
Penrose Drain	73	20 (27.4)	9 (20.5)	11 (37.9)	0.17	10 (21.3)	10 (38.5)	0.19	12 (21.8)	8 (44.4)	0.08
Fulminant NEC, n (%)	75	11 (14.7)	8 (17.4)	3 (10.3)	0.52	8 (16.3)	3 (11.5)	0.74	8 (14.0)	3 (16.7)	0.72
Length of bowel resected (cm, median [IQR])	75	22.9 [11.9;37.2]	26.0 [12.7;54.1]	22.3 [10.4;28.8]	0.1	28.0 [12.7;45.3]	21.4 [10.6;26.1]	0.08	23.0 [12.7;40.0]	17.8 [10.3;29.4]	0.17
Length of Jejunum Lost (cm, median [IQR])	69	0.00 [0.00;12.5]	2.90 [0.00;26.5]	0.00 [0.00;4.75]	0.05	1.45 [0.00;24.1]	0.00 [0.00;5.25]	0.1	0.00 [0.00;16.2]	0.00 [0.00;6.18]	0.34
Length of Ileum Lost (cm, median [IQR])	73	8.00 [0.00;20.9]	6.40 [0.00;19.0]	9.00 [2.75;21.4]	0.24	7.15 [0.00;24.0]	8.50 [2.00;20.5]	0.48	7.15 [0.00;23.2]	8.50 [2.00;20.5]	0.68
Length of Colon Lost (cm, median [IQR])	73	0.00 [0.00;10.6]	0.00 [0.00;14.2]	0.00 [0.00;2.90]	0.89	0.00 [0.00;13.1]	0.00 [0.00;2.70]	0.97	0.00 [0.00;11.6]	0.00 [0.00;2.08]	0.88
Region of Bowel Resected, n (%)	74				0.99			0.99			0.98
Large Bowel or Both		31 (41.9)	19 (42.2)	12 (41.4)		20 (41.7)	11 (42.3)		24 (42.9)	7 (38.9)	
Small bowel		43 (58.1)	26 (57.8)	17 (58.6)		28 (58.3)	15 (57.7)		32 (57.1)	11 (61.1)	
Presence of Ileocecal Valve, n (%)	74	44 (59.5)	25 (55.6)	19 (65.5)	0.54	26 (54.2)	18 (69.2)	0.31	31 (55.4)	13 (72.2)	0.32
Type of Stoma											
Jejunostomy, n (%)	75	25 (33.3)	14 (30.4)	11 (37.9)	0.68	15 (30.6)	10 (38.5)	0.67	18 (31.6)	7 (38.9)	0.77
Ileostomy, n (%)	75	37 (49.3)	22 (47.8)	15 (51.7)	0.93	25 (51.0)	12 (46.2)	0.87	30 (52.6)	7 (38.9)	0.46
Colostomy, n (%)	75	5 (6.67)	1 (2.17)	4 (13.8)	0.7	1 (2.04)	4 (15.4)	0.046	2 (3.51)	3 (16.7)	0.09
Total Small Bowel Lost, cm (mean +/− SD)	75	14.9 [8.05;31.5]	16.9 [8.20;42.9]	11.0 [8.00;22.3]	0.09	16.9 [8.10;40.0]	11.0 [6.73;21.8]	0.06	16.2 [8.00;37.0]	10.7 [8.35;21.8]	0.148
Residual Small Bowel, cm (mean +/− SD)	75	86.6 [64.4;99.3]	83.4 [64.3;99.7]	89.0 [75.7;98.5]	0.58	83.1 [62.0;98.7]	89.9 [76.1;99.6]	0.37	86.6 [64.1;98.7]	87.0 [75.7;99.6]	0.678
Residual Colon, cm (mean +/− SD)	75	24.4 [21.6;27.8]	24.4 [21.9;36.5]	22.7 [21.5;24.4]	0.23	24.4 [21.6;32.7]	23.0 [21.7;24.4]	0.37	24.4 [21.5;27.8]	23.5 [22.3;27.0]	0.574
**Histopathological Factors**											
Necrosis (≥ 50 vs < 50%, median [IQR])	75	2.00 [1.00;3.00]	2.00 [1.00;3.00]	2.00 [1.00;3.00]	0.13	2.00 [1.00;3.00]	1.50 [0.25;3.00]	0.11	2.00 [1.00;3.00]	1.00 [0.25;2.00]	0.05
Inflammation (≥ 50 vs < 50%, median [IQR])	75	2.00 [2.00;3.00]	2.00 [1.00;3.00]	2.00 [2.00;3.00]	0.61	2.00 [1.00;3.00]	2.00 [2.00;2.75]	0.72	2.00 [2.00;3.00]	2.00 [2.00;2.75]	0.77
Hemorrhage (≥ 50 vs < 50%, median [IQR])	75	2.00 [2.00;4.00]	3.00 [2.00;4.00]	2.00 [2.00;3.00]	0.11	3.00 [2.00;4.00]	2.00 [1.25;3.00]	0.09	3.00 [2.00;4.00]	2.00 [2.00;3.00]	0.41
Reparative change (≥ 50 vs < 50%, median [IQR])	75	0.00 [0.00;1.00]	0.00 [0.00;1.00]	1.00 [0.00;1.00]	**0.004**	0.00 [0.00;1.00]	1.00 [0.00;1.00]	**0.002**	0.00 [0.00;1.00]	1.00 [0.00;1.00]	**0.013**
Necrosis, n (%)	75				0.54			0.44			0.35
**0%**		13 (17.3)	6 (13.0)	7 (24.1)		6 (12.2)	7 (26.9)		8 (14.0)	5 (27.8)	
**25%**		14 (18.7)	7 (15.2)	7 (24.1)		8 (16.3)	6 (23.1)		9 (15.8)	5 (27.8)	
25–50%		19 (25.3)	13 (28.3)	6 (20.7)		14 (28.6)	5 (19.2)		15 (26.3)	4 (22.2)	
50–75		23 (30.7)	16 (34.8)	7 (24.1)		17 (34.7)	6 (23.1)		20 (35.1)	3 (16.7)	
>75		6 (8.00)	4 (8.70)	2 (6.90)		4 (8.16)	2 (7.69)		5 (8.77)	1 (5.56)	
Inflammation, n (%)	75				0.1			0.11			0.35
**0**		3 (4.00)	3 (6.52)	0 (0.00)		3 (6.12)	0 (0.00)		3 (5.26)	0 (0.00)	
**25**		14 (18.7)	10 (21.7)	4 (13.8)		10 (20.4)	4 (15.4)		11 (19.3)	3 (16.7)	
25–50		33 (44.0)	16 (34.8)	17 (58.6)		18 (36.7)	15 (57.7)		23 (40.4)	10 (55.6)	
50–75		18 (24.0)	14 (30.4)	4 (13.8)		15 (30.6)	3 (11.5)		16 (28.1)	2 (11.1)	
> 75		7 (9.33)	3 (6.52)	4 (13.8)		3 (6.12)	4 (15.4)		4 (7.02)	3 (16.7)	
Hemorrhage, n (%)	75				**0.019**			**0.012**			0.37
**0**		5 (6.67)	5 (10.9)	0 (0.00)		5 (10.2)	0 (0.00)		5 (8.77)	0 (0.00)	
**25**		9 (12.0)	2 (4.35)	7 (24.1)		2 (4.08)	7 (26.9)		6 (10.5)	3 (16.7)	
25–50		24 (32.0)	13 (28.3)	11 (37.9)		15 (30.6)	9 (34.6)		16 (28.1)	8 (44.4)	
50–75		15 (20.0)	9 (19.6)	6 (20.7)		9 (18.4)	6 (23.1)		11 (19.3)	4 (22.2)	
> 75		22 (29.3)	17 (37.0)	5 (17.2)		18 (36.7)	4 (15.4)		19 (33.3)	3 (16.7)	
Reparative Change, n (%)	75	31 (41.3)	13 (28.3)	18 (62.1)	**0.008**	14 (28.6)	17 (65.4)	**0.005**	5 (8.77)	0 (0.00)	**0.026**
**Sepsis Variables**											
Central Line Present (days, median [IQR])	62	57.0 [31.2;77.8]	57.0 [30.0;65.0]	59.0 [41.0;97.5]	0.16	54.5 [26.2;64.8]	68.0 [43.8;100]	**0.044**	54.5 [30.0;66.0]	68.0 [40.0;97.8]	0.19
Positive Blood Culture Sepsis, n (%)	75	24 (32.0)	13 (28.3)	11 (37.9)	0.54	14 (28.6)	10 (38.5)	0.54	17 (29.8)	7 (38.9)	0.67
Antibiotic Duration (days, median [IQR])	60	8.00 [5.00;14.0]	7.00 [3.50;13.5]	10.0 [7.00;14.0]	0.23	8.00 [4.25;13.8]	8.50 [7.00;14.0]	0.45	8.00 [5.00;14.0]	8.00 [7.00;14.5]	0.42
CRP 24 Hours After NEC (median [IQR])	56	14.2 [4.20;19.8]	14.4 [3.90;18.5]	13.6 [5.35;21.3]	0.68	14.2 [4.20;18.8]	14.4 [5.60;22.1]	0.61	13.8 [4.00;19.4]	15.6 [7.88;20.6]	0.57
CRP 48 Hours After NEC (median [IQR])	51	15.5 [5.70;22.0]	15.5 [3.30;21.2]	16.1 [7.30;24.3]	0.46	15.6 [3.30;21.4]	15.4 [7.10;23.0]	0.8	15.7 [4.70;22.6]	14.9 [6.90;21.0]	0.68
CRP 96 Hours After NEC (median [IQR])	47	8.30 [4.30;20.0]	7.70 [4.10;14.3]	13.3 [5.00;22.7]	0.37	8.30 [4.10;15.5]	10.8 [4.80;21.8]	0.68	8.40 [5.13;20.4]	8.20 [3.80;18.2]	0.51
CRP 1 Week After NEC (median [IQR])	47	6.50 [3.00;13.8]	5.10 [3.00;7.35]	10.4 [4.68;17.6]	0.06	5.35 [3.05;7.62]	8.60 [4.05;17.2]	0.15	5.90 [3.40;11.2]	7.25 [1.78;13.8]	0.75
CRP 2 Weeks After NEC (median [IQR])	48	4.05 [1.82;7.20]	3.70 [1.60;7.20]	4.50 [2.00;7.20]	0.49	3.70 [1.60;8.10]	4.50 [2.00;6.50]	0.67	4.05 [2.08;6.75]	3.80 [1.48;7.20]	0.73

Categorical variables are presented as count (column percentage). The presence of bold values signifies p < 0.05. Continuous variables are presented as mean (standard deviation) if normality has been met and, if not normally distributed, as median with interquartile range.

**Table 3:** Postoperative Clinical Outcomes in infants with surgical NEC Receiving invention at < or > 48, 96, 168 hour time points

	48 hours					96 hours			168 Hrs		
	n	n = 75	≤ 48 hours n = 46	> 48 hours n = 29	*p*	≤ 96 hours n = 49	> 96 hours n = 26	*p*	≤ 168 hrs n = 57	> 168 hrs n = 18	*p*
**Post Operative Intestinal Features**											
Blood Transfusion 48 hours After NEC onset, n (%)	68	51 (75.0)	32 (80.0)	19 (67.9)	0.39	34 (79.1)	17 (68.0)	0.47	38 (76.0)	13 (72.2)	0.76
Blood Transfusion 2 days to 2 Weeks After NEC onset, (median [IQR])	66	3.00 [2.00;5.00]	3.00 [2.00;5.00]	3.50 [2.00;5.00]	0.85	3.00 [2.00;5.00]	3.00 [2.00;5.00]	0.89	4.00 [2.00;5.00]	3.00 [2.00;4.00]	0.24
Platelet Transfusion 48 hours After NEC, n (%)	62	28 (45.2)	17 (45.9)	11 (44.0)	0.99	17 (42.5)	11 (50.0)	0.76	21 (44.7)	7 (46.7)	0.99
Post Operative Ileus (days, median [IQR])	58	19.6 (19.5)	20.3 (18.0)	18.7 (21.8)	0.77	20.1 (17.7)	18.9 (22.2)	0.83	20.9 (19.5)	16.4 (19.7)	0.43
Duration of Parenteral Nutrition (days, median [IQR])	74	82.0 [38.2;120]	70.0 [25.8;114]	91.5 [67.0;144]	0.11	65.0 [22.0;112]	109 [76.0;147]	0.011	81.5 [29.2;115]	96.5 [70.2;136]	0.24
Postoperative Day at Starting Enteral Feedings (days), n (%)	59	14.0 [11.5;20.5]	14.0 [11.5;24.5]	14.0 [11.8;16.2]	0.46	14.0 [11.8;23.8]	14.0 [11.5;16.5]	0.46	15.0 [11.5;23.5]	13.0 [11.8;15.0]	0.18
Time to Reach Full Feeds, (days) (median [IQR])	51	70.0 [29.0;96.0]	65.0 [30.0;106]	70.5 [27.2;84.2]	0.58	62.5 [30.5;104]	71.0 [27.0;87.0]	0.68	73.5 [32.5;104]	61.0 [20.0;74.0]	0.19
Cholestasis, n (%)	67	45 (67.2)	23 (59.0)	22 (78.6)	0.16	25 (59.5)	20 (80.0)	0.15	29 (59.2)	16 (88.9)	**0.05**
Surgical Complication/Morbidity, n (%)	75	23 (30.7)	11 (23.9)	12 (41.4)	0.18	12 (24.5)	11 (42.3)	0.18	16 (28.1)	7 (38.9)	0.57
Wound Dehiscence, n (%)	75	9 (12.0)	5 (10.9)	4 (13.8)	0.73	5 (10.2)	4 (15.4)	0.71	8 (14.0)	1 (5.56)	0.68
Wound Infection, n (%)	75	6 (8.00)	3 (6.52)	3 (10.3)	0.67	3 (6.12)	3 (11.5)	0.41	4 (7.02)	2 (11.1)	0.63
Stricture, n (%)	75	5 (6.67)	3 (6.52)	2 (6.90)	0.99	3 (6.12)	2 (7.69)	0.99	3 (5.26)	2 (11.1)	0.59
Adhesions, n (%)	75	7 (9.33)	2 (4.35)	5 (17.2)	0.1	2 (4.08)	5 (19.2)	**0.045**	4 (7.02)	3 (16.7)	0.35
**Postoperative Systemic Course**											
Assisted Ventilation (Intubated), n (%)	72				**0.03**			**0.042**			0.13
Intubated (Invasive ventilation)		62 (86.1)	34 (79.1)	28 (96.6)		37 (80.4)	25 (96.2)		45 (83.3)	17 (94.4)	
High-flow		5 (6.94)	5 (11.6)	0 (0.00)		5 (10.9)	0 (0.00)		5 (9.26)	0 (0.00)	
CPAP		1 (1.39)	0 (0.00)	1 (3.45)		0 (0.00)	1 (3.85)		0 (0.00)	1 (5.56)	
Room air		4 (5.56)	4 (9.30)	0 (0.00)		4 (8.70)	0 (0.00)		4 (7.41)	0 (0.00)	
24 h Ionotropic support, n (%)	75	59 (78.7)	36 (78.3)	23 (79.3)	0.99	39 (79.6)	20 (76.9)	0.99	47 (82.5)	12 (66.7)	0.19
AKI by Serum Creatinine, n (%)	75	44 (58.7)	22 (47.8)	22 (75.9)	**0.03**	25 (51.0)	19 (73.1)	0.11	32 (56.1)	12 (66.7)	0.61
AKI by Urine Output, n (%)	63	36 (57.1)	22 (57.9)	14 (56.0)	0.99	24 (58.5)	12 (54.5)	0.97	28 (58.3)	8 (53.3)	0.97

Categorical variables are presented as count (column percentage). The presence of bold values signifies p < 0.05. Continuous variables are presented as mean (standard deviation) if normality has been met and, if not normally distributed, as median with interquartile range.

**Table 4:** Clinical Outcomes in infants with surgical NEC receiving invention at < or > 48, 96, 168 hour time points

	48 hours					96 hours			168 Hrs		
	n	n = 75	≤ 48 hours n = 46	> 48 hours n = 29	*p*	≤ 96 hours n = 49	> 96 hours n = 26	*p*	≤ 168 hrs n = 57	> 168 hrs n = 18	*p*
**Outcomes**											
MRI Corrected Gestational Age (weeks, median [IQR])	33	40.3 [38.1;46.1]	41.7 [38.3;45.9]	39.2 [38.2;46.2]	0.62	41.7 [38.3;45.9]	39.2 [38.2;46.2]	0.62	41.3 [38.2;47.3]	38.8 [38.2;40.0]	0.4
White Matter Abnormality, n (%)	34				0.46			0.46			0.2
Normal		12 (35.3)	7 (30.4)	5 (45.5)		7 (30.4)	5 (45.5)		8 (29.6)	4 (57.1)	
Abnormal		22 (64.7)	16 (69.6)	6 (54.5)		16 (69.6)	6 (54.5)		19 (70.4)	3 (42.9)	
White Matter Abnormality, n (%)	34				0.58			0.58			0.6
Normal		12 (35.3)	7 (30.4)	5 (45.5)		7 (30.4)	5 (45.5)		8 (29.6)	4 (57.1)	
Mild		10 (29.4)	6 (26.1)	4 (36.4)		6 (26.1)	4 (36.4)		9 (33.3)	1 (14.3)	
Moderate		8 (23.5)	7 (30.4)	1 (9.09)		7 (30.4)	1 (9.09)		7 (25.9)	1 (14.3)	
Severe		4 (11.8)	3 (13.0)	1 (9.09)		3 (13.0)	1 (9.09)		3 (11.1)	1 (14.3)	
Grey Matter Abnormality, n (%)	34				0.99			0.99			1
Normal		30 (88.2)	20 (87.0)	10 (90.9)		20 (87.0)	10 (90.9)		24 (88.9)	6 (85.7)	
Abnormal		4 (11.8)	3 (13.0)	1 (9.09)		3 (13.0)	1 (9.09)		3 (11.1)	1 (14.3)	
BPD, n (%)	54				0.93			0.47			0.7
Normal		6 (11.1)	4 (12.1)	2 (9.52)		5 (14.3)	1 (5.26)		6 (14.0)	0 (0.00)	
Mild		10 (18.5)	7 (21.2)	3 (14.3)		8 (22.9)	2 (10.5)		8 (18.6)	2 (18.2)	
Moderate		11 (20.4)	6 (18.2)	5 (23.8)		6 (17.1)	5 (26.3)		9 (20.9)	2 (18.2)	
Severe		27 (50.0)	16 (48.5)	11 (52.4)		16 (45.7)	11 (57.9)		20 (46.5)	7 (63.6)	
Postnatal Use of Steroids, n (%)	75	48 (64.0)	30 (65.2)	18 (62.1)	0.98	32 (65.3)	16 (61.5)	0.94	36 (63.2)	12 (66.7)	1
Type of Steroid Used, n (%)	48				0.4			0.2			1
Hydrocortisone		41 (85.4)	27 (90.0)	14 (77.8)		29 (90.6)	12 (75.0)		31 (86.1)	10 (83.3)	
Dexamethasone		7 (14.6)	3 (10.0)	4 (22.2)		3 (9.38)	4 (25.0)		5 (13.9)	2 (16.7)	
Discharge Weight (g, median [IQR])	73	3625 [2590;4850]	3513 [2492;4529]	4130 [2800;5085]	0.29	3545 [2520;4842]	4065 [2725;4816	0.57 ]	3606 [2520;4842]	4210 [2852;4816]	0.4
Length of Stay (days, median [IQR])	75	128 [71.0;174]	130 [66.8;180]	125 [90.0;171]	0.93	117 [56.0;178]	140 [97.5;171]	0.39	117 [69.0;178]	140 [90.2;170]	0.7
Death, n (%)	75	25 (33.3)	13 (28.3)	12 (41.4)	0.36	16 (32.7)	9 (34.6)	0.99	19 (33.3)	6 (33.3)	1

Categorical variables are presented as count (column percentage). The presence of bold values signifies p < 0.05. Continuous variables are presented as mean (standard deviation) if normality has been met and, if not normally distributed, as median with interquartile range.
